# Electrospun patterned porous scaffolds for the support of ovarian follicles growth: a feasibility study

**DOI:** 10.1038/s41598-018-37640-1

**Published:** 2019-02-04

**Authors:** Liliana Liverani, Nathalie Raffel, Amir Fattahi, Alexander Preis, Inge Hoffmann, Aldo R. Boccaccini, Matthias W. Beckmann, Ralf Dittrich

**Affiliations:** 1 Institute of Biomaterials, Department of Materials Science and Engineering, University of Erlangen-Nuremberg, 91058 Erlangen, Germany; 2 Department of Obstetrics and Gynecology, Erlangen University Hospital, Friedrich-Alexander-Universität Erlangen–Nürnberg, Comprehensive Cancer Center ER-EMN, 91054 Erlangen, Germany

## Abstract

Recently, the interest of the scientific community is focused on the application of tissue engineering approach for the fertility restoration. In this paper innovative patterned electrospun fibrous scaffolds were fabricated and used as 3D system for porcine follicles culture. The obtained scaffolds demonstrated to be a suitable support which did not alter or interfere with the typical spherical follicles morphology. The fibrillar structure of the scaffolds mimics the morphology of the healthy native tissue. The use of porcine follicles implied many advantages respect to the use of mouse model. Relevant results showed that more than the scaffold pattern and struts dimension, the selection of proper biomaterials improve the follicles adhesion and development.

## Introduction

Cancer statistics show a continuous increase in newly diagnosed cases all over the world in the last decades. The same trend has been reported for the diagnosis of cancer in paediatric patients. Because of the improvement in the early diagnosis and the enhancement in treatment, also the statistical data about the rate of cancer survivors reported positive and encouraging results on the life expectancy of the patients. Actually, for paediatric cancer the five-year survival rate approaches 80%^1^. In particular, a diagnosis of cancer during childhood and adolescence introduces major challenges regarding treatment-related late effects, and as survival rates improve, there is much attention on interventions to improve the quality of life during survivorship^1–3^.

In this context, the process of fertility preservation after the exposure to gonadotoxic therapeutic agents in oncological patients and in particular the ovarian tissue cryopreservation has been the focus of many research papers and practise committee opinions in the last years^4–9^. Among the other techniques aiming to preserve the reproductive potential in oncological patients, ovarian tissue cryopreservation is currently considered an innovative or established method^10–12^, representing an option for patients who require immediate gonadotoxic treatment of aggressive malignancies when there is not enough time to allow the woman to undergo ovulation induction, oocytes retrieval and cryopreservation of oocytes and/or embryos. Moreover, ovarian tissue cryopreservation is the only option available for fertility preservation in young girls in prepubertal age or in women affected by hormone-sensitive malignancies^1,4,13,14^. Other potential indications for this fertility preservation technique are related to patients with genetic mutations that pose a high risk for premature ovarian failure and are not eligible for other fertility preservation approaches^4,7^. Unfortunately, for the cancer patients with moderate-to-high risk of ovarian metastasis, even ovarian tissue cryopreservation and implantation after treatment is not possible, due to the possibility of reimplanting malignant cells and recurrence of the cancer. So isolation of the follicles from ovarian tissue and then reimplantation of isolated follicles in the form of artificial ovary can ensure that no malignant cells will be returned back to the patient^15^.

The ideal artificial ovary should mimic the native organ in terms of environmental conditions for the follicles growth and maturation, providing the appropriate mechanical support which allows the preservation of follicles spheroidal structure. It should also have a specific architecture with pore morphology to improve follicles adhesion, allowing the exchange of nutrients and metabolic products and promoting the vascularization. Moreover, it should also provide a support to mimic the native tissue consistency during the reimplantation surgery, facilitating the surgical procedure^16^.

Recently, the introduction of the definition of reproductive tissue engineering (REPROTEN) for the application of tissue engineering concept to fertility restoration and improvement the quality of life of patients with reproductive disfunctions demonstrates the increased interest on this topic^17^.

Among the different techniques for scaffold fabrication, the electrospinning allows the fabrication of fibers, mimicking the fibrillar morphology of the native extra cellular matrix. The electrospinning technique is based on the application of a high electric potential between two electrodes of opposite polarities. This high voltage is able to overcome the surface tension inside a polymeric solution allowing the complete evaporation of the solvent and the formation of fibrous structure on a grounded collector^18^. The electrospinning is versatile because it allows the use and the processability of a huge number of natural and synthetic polymers and their blends, the possibility to select different type of solvents, with particular focus in the recent research works on the use of benign solvents and on green electrospinning^19,20^.

One disadvantage of the electrospun mats is represented by the density of the mats which could limit cells infiltration inside the scaffold. For this reason, electrospun fibers with macropores with average size of 0.3 mm, previously developed by the same authors^21^, were used in the present work to allow the preservation of follicular spheroidal structure.

In the present study, poly(epsilon caprolactone) (PCL) and a blend of it with gelatin were used for the scaffolds fabrication. The use of PCL is widely reported for several applications in biomedical (i.e. tissue engineering), food and other industrial fields^22–24^, but not yet investigated in literature for REPROTEN purposes. PCL is a biodegradable polyester and in aqueous environment it shows low degradation rate with harmless byproducts and its properties could be modulated by blending PCL with other polymers^25–27^. In particular, the use of PCL blends with gelatin, a natural polymer derived from collagen already investigated for artificial ovary applications^28^, offers the advantage to reduce the PCL hydrophobicity, improving biocompatibility, in terms of cells response^29–31^. The processing of PCL, other polyesters and their blends with the electrospinning is well known and documented in literature in the last decades. Since the beginning of the spreading of the electrospinning technique, PCL, other polyesters and their blends have been processed by this method^32,33^. The solvents systems commonly used to solve PCL for the electrospinning process are based on chloroform, dichloromethane, N,N-dimethylformamide, tetrahydrofuran and their mixture with methanol and toluene^34–37^. Most of the solvents listed above even if appropriate for the process, they show high toxicity and require additional post treatments for their removal from the obtained electrospun mats for biomedical applications. On this basis, the focus of numerous recent researches was the optimization of electrospinning using benign solvents, *i*.*e*. formic acid, acetic acid, and acetone^38–41^.

In the present work, innovative patterned fibrous scaffolds fabricated by using PCL and PCL/gelatin were fabricated, characterized and investigated as suitable substrate to support porcine follicles adhesion and growth. In particular, porcine follicles isolated from porcine ovaries were used in this work since they represent the optimal compromise of performing *in vitro* tests on large animals with the length of the follicle maturation period more comparable to humans compared to the murine model, having less ethical concerns.

## Results

### Electrospun scaffolds fabrication

The relevance of the use of biomaterials for the support of three dimensional follicles culture has been highlighted in a recent scientific publication of Laronda *et al*.^28^ in which it was reported the use of 3D printed gelatin hydrogel scaffolds to restore ovarian function in sterilized mice. Another recent study highlights the relevance on a biomimetic approach in the scaffolds fabrication in order to replicate the structure and morphology of native human ovarian tissue, by using fibrin clots^42^. In literature, the use of 3D culture system for culturing follicles by using microdrops or hydrogel-based system has been already reported^43^. The advantage of 3D culture systems is that they mimic closely the physiological environment of the ovary, preserving follicular architecture and the interaction between somatic and germ cells^43^.

**Figure 1:**
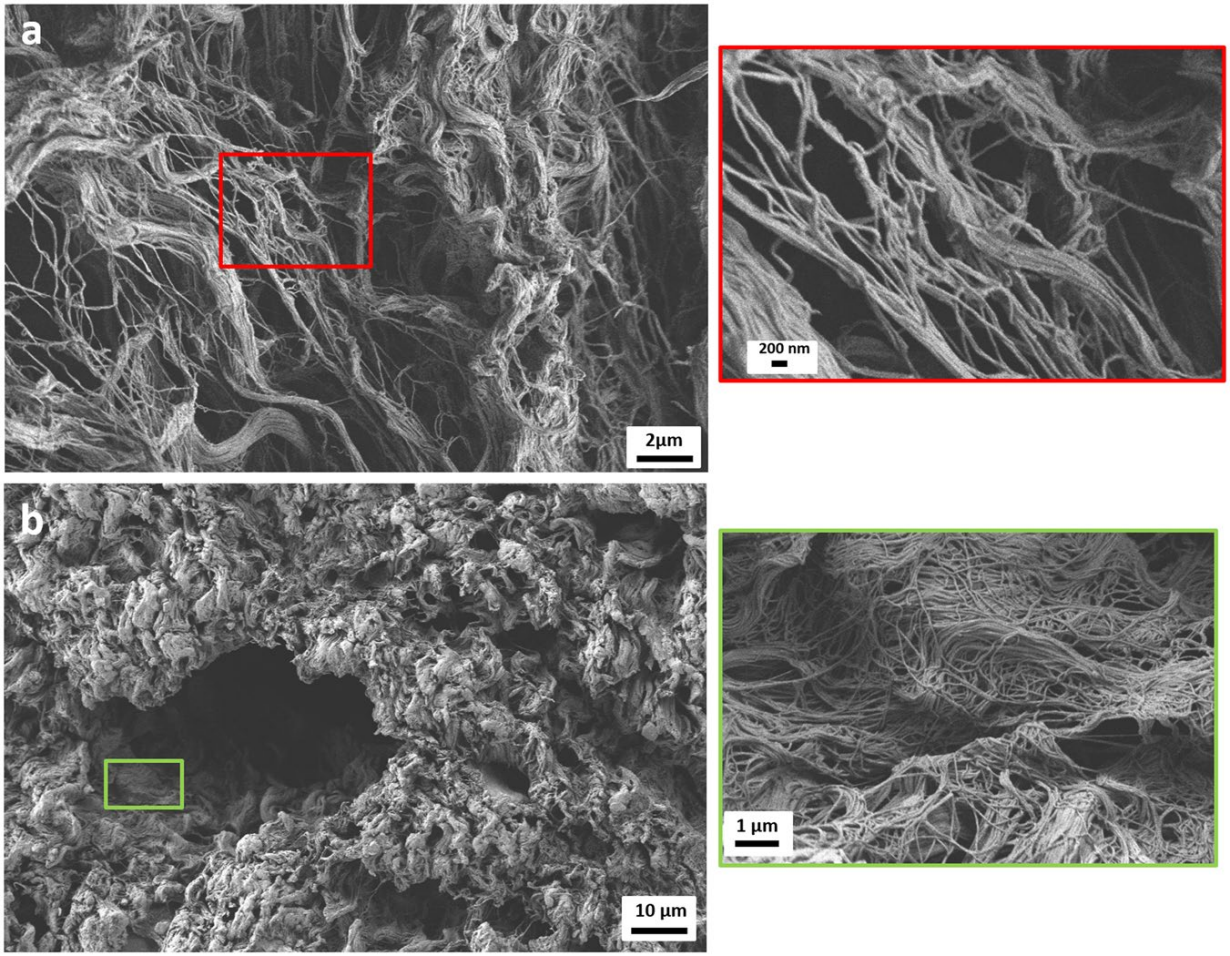
SEM micrographs of cortex (**a**) and medulla (**b**) from porcine ovarian tissue. The magnifications are: 10000X (a) scale bar 2 µm, and in its inset 45000X, scale bar 200 nm. For the medulla (**b**) magnification is 2000X, scale bar 10 µm and in the inset 20000X scale bar 1 µm.

The biomimetic approach used in this paper aims to reproduce the human native healthy tissue in its morphology. Morphological analysis performed by using SEM technique on human ovarian cortex was already reported in literature by Chiti *et al*.^42^. It is possible to notice that the nanofibrillary structure of the native tissue could inspire the design of scaffolds for REPROTEN applications. In the present work, considering the use of porcine ovarian follicles, the morphological analysis of decellularized porcine ovarian tissue (both cortex and medulla) was performed and reported in Fig. 1. The presence of fibrillary morphology in both the analyzed tissues confirmed the relevance of using the electrospinning technique for the fabrication of biomimetic scaffolds.

**Figure 2:**
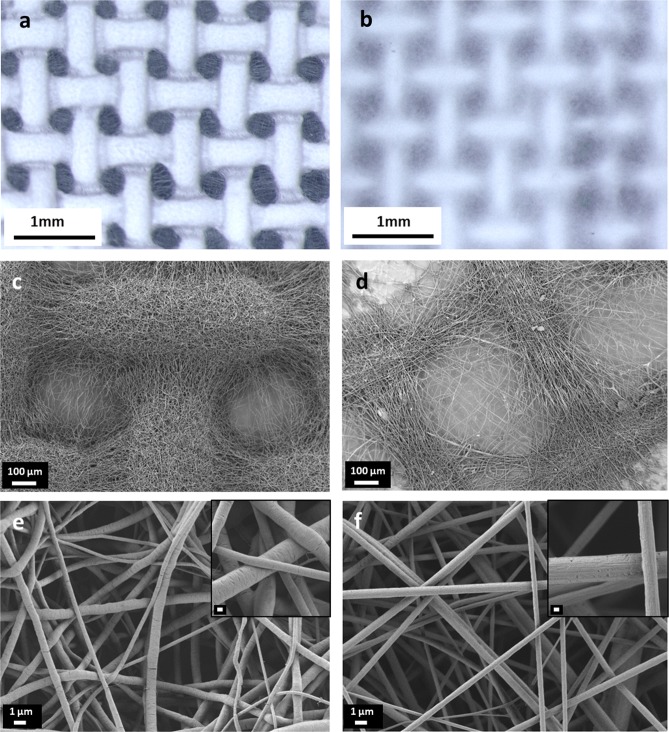
Light microscopy images and SEM micrographs of neat PCL (**a**,**c**,**e**) and PCL/gel (**b**,**d**,**f**) patterned electrospun scaffolds. For SEM micrographs, the magnifications are: 200X (**c**,**d**) scale bar 100 µm, 10000X (**e**,**f**) scale bar 1 µm and in the insets of (**e** and **f**) the magnification of 45000X with scale bar of 200 nm.

In the present work, 3D macroporous electrospun scaffolds were obtained for neat PCL and blend PCL/gelatin (PCL/gel) as reported in Fig. 2. The measured average fiber diameter for neat PCL was 1096 ± 764 nm and for PCL/gel samples was 495 ± 133 nm. The average fibers diameter of neat PCL is slightly different from the literature, because of the effect of the specific metal target for the collection of the macroporous nets. The calculated values for the average pore area was 3.4 10^−2^ mm^2^ for neat PCL and 2.2 10^−4^ mm^2^ for the blended samples.

**Figure 3:**
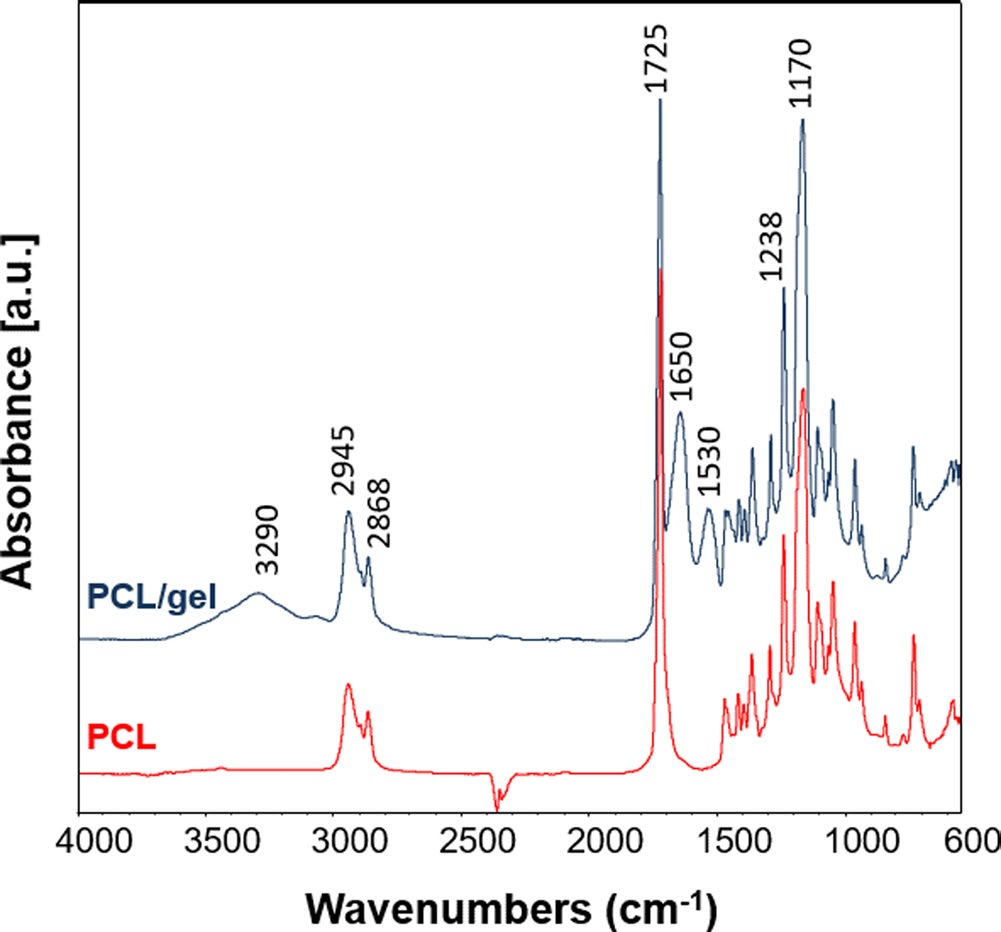
ATR-FTIR spectra of PCL and PCL/gel electrospun nets analyzed between 4000 and 600 cm^−1^. The main peaks explained in the text are also reported on the spectra (both bands related to PCL and gelatin contributions are indicated on PCL/gel spectrum).

ATR-FTIR was performed to investigate any possible effects of the electrospinning process on PCL nets and to confirm the presence of PCL and gelatin in the blended sample. Characteristic peaks for PCL were detected in neat PCL sample, confirming that the electrospinning process does not affect the polymer structure (Fig. 3). In particular the main characteristic peaks are 2945 cm^−1^ and 2868 cm^−1^ indicating the asymmetric and symmetric stretching of CH_2_, at 1725 cm^−1^ the stretch of the C=O bond in ester groups and at 1238 cm^−1^ and 1170 cm^−1^ the asymmetric and symmetric C-O-C stretching^44^. In the spectrum of PCL/gel sample besides the main peaks ascribable to PCL, additional bands related to the presence of gelatin were detected, like the broad peak centered around 3290 cm^−1^, due to amide A, the peaks at 1650 cm^−1^ and 1530 cm^−1^ due to amide I and amide II, respectively^44^.

### Scaffolds degradation studies

The effects of the degradation after immersion in Phosphate Buffered Saline (PBS) and follicles medium were investigated for both PCL and PCL/gel samples, in particular for what concerns possible alterations of fibrillary morphology and the chemical composition. Additionally, also possible pH variations were investigated, but for both samples (neat PCL and PCL/gel) and both media, no significant differences were measured. Measured pH values were stable till the last timepoint after 30 days of immersion and comparable with the control (medium without scaffolds), confirming the suitability of the scaffolds for follicles culture. The measured values and their statistical analysis are reported in Table 1.

**Table 1:** Average measured pH values of PCL and PCL/gel net scaffolds immersed in medium for 1, 10 and 30 days (control was medium without scaffolds).

Culture duration	Control (without scaffold)	PCL net	PCL/gel net
1 day	7.44 ± 0.03	7.39 ± 0.02	7.47 ± 0.06
10 days	6.79 ± 0.01^*^	6.69 ± 0.02^a,*^	6.74 ± 0.01^a,b,*^
30 days	7.30 ± 0.01^*,$^	7.31 ± 0.01^*,$^	7.29 ± 0.00^*,$^

ANOVA with post-hoc Tukey was used for statistical analysis.

Significant difference (*p* < 0.05) in comparison with ^a^Control and ^b^PCL net in the same culture duration.

Significant difference (*p* < 0.05) in comparison with ^*^1 day and ^$^10-days culturing in the same culture condition.

**Figure 4:**
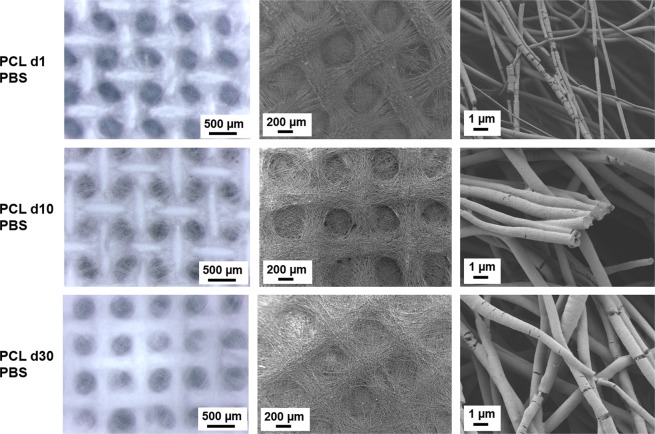
Light microscope images and SEM micrographs of the PCL electrospun nets after immersion in PBS for 1 day, 10 days and 30 days at different magnifications. For light microscope images the magnification is 4x for all samples. For SEM micrographs, the magnifications are: 100X scale bar 200 µm, and 10000X scale bar 1 µm.

**Figure 5:**
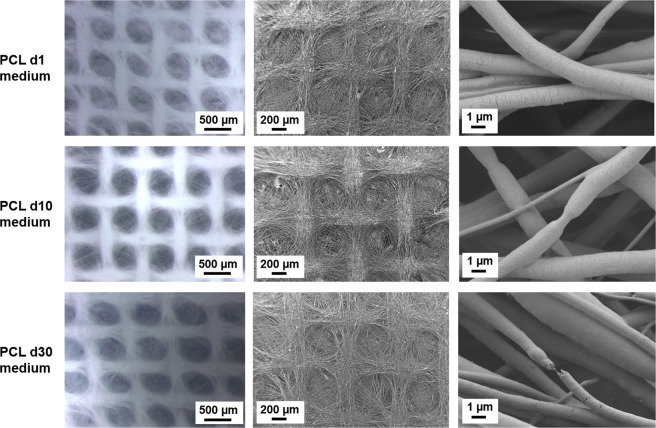
Light microscope images and SEM micrographs of the PCL electrospun nets after immersion in medium for 1 day, 10 days and 30 days at different magnifications. For light microscope images the magnification is 4x for all samples. For SEM micrographs, the magnifications are: 100X scale bar 200 µm, and 20000X scale bar 1 µm.

**Figure 6:**
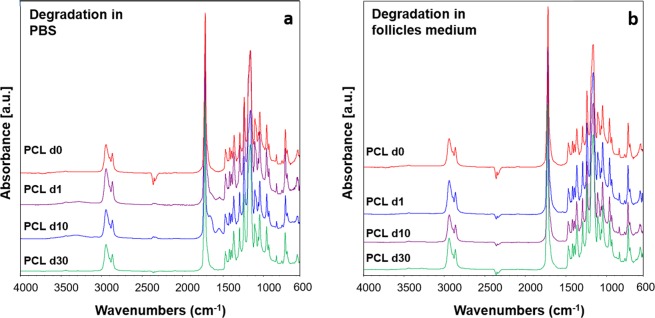
ATR-FTIR spectra of PCL electrospun nets before (PCL d0) and after immersion in PBS (**a**) and before and after immersion in follicles medium (**b**) for 1 (PCL d1), 10 (PCL d10), and 30 days (PCL d30), analyzed between 4000 and 600 cm^−1^.

PCL nets did not show any significant changes in the macropores structure and in the fibrillary morphology through all the time points in both media, as showed in Figs 4 and 5. It is interesting to observe that, even if the pore structure and the fibrous morphology were preserved, in PBS a faster degradation of the PCL fibers surface occurred, already after one day of immersion in PBS. Similar degradation was observed on PCL fibers after 30 days of immersion in the follicles medium. The explanation for this fibers surface degradation is related to the presence of micrometric cracks on the fibers surface due to the solvent evaporation during the electrospinning process. In correspondence of these points, it is possible to notice the effects of fibers surface degradation. ATR-FTIR spectra before and after all the timepoints, reported in Fig. 6a,b do not show any modifications of PCL after the immersion in both media.

**Figure 7:**
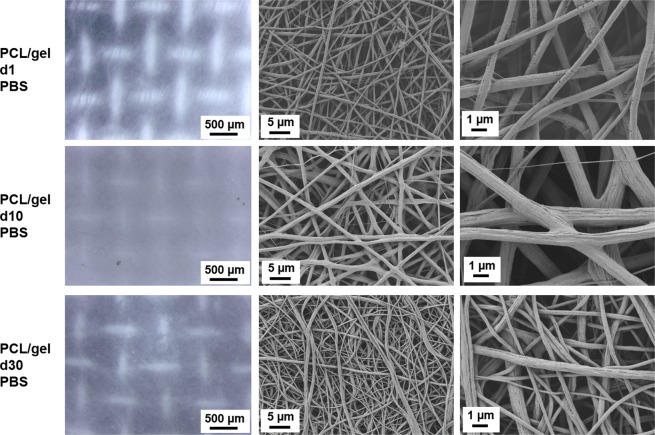
Light microscope images and SEM micrographs of the PCL/gel electrospun nets after immersion in PBS for 1 day, 10 days and 30 days at different magnifications. For light microscope images the magnification is 4x for all samples. For SEM micrographs, the magnifications are: 5000X scale bar 5 µm, and 20000X scale bar 1 µm.

**Figure 8:**
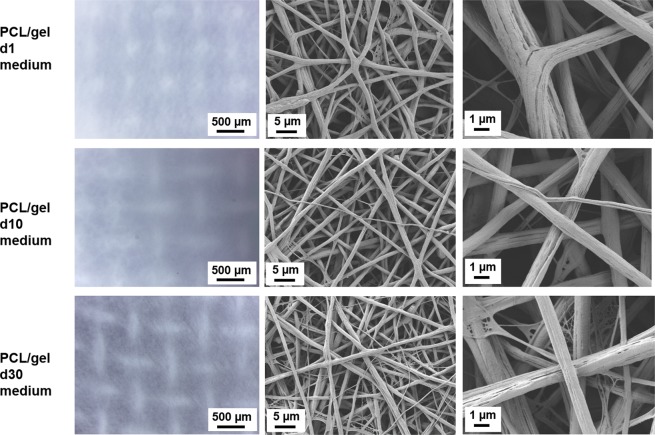
Light microscope images and SEM micrographs of the PCL/gel electrospun nets after immersion in medium for 1 day, 10 days and 30 days at different magnifications. For light microscope images the magnification is 4x for all samples. For SEM micrographs, the magnifications are: 5000X scale bar 5 µm, and 20000X scale bar 1 µm.

For PCL/gel samples the presence of macropores could be noticed only on the light microscope images, as showed in Figs 7 and 8. Additionally, a change in the fibers surface occurred already after the first time point, as showed in Figs 7 and 8. In fact, some straight roughness appeared on the fibers surface, which could be related to a release of gelatin from the blended fiber surface, but on the contrary of the neat PCL samples, in this case no evident broken fibers are visible at any time points for both media.

**Figure 9:**
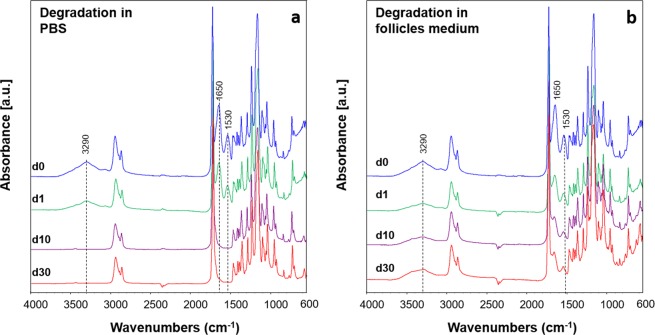
ATR-FTIR spectra of PCL/gel electrospun nets before (d0) and after immersion in PBS (**a**) and follicles medium (**b**) for 1 (d1), 10 (d10) and 30 (d30) days, analyzed between 4000 and 600 cm^−1^.

For what concerns the blended fibers composition, ATR-FTIR analysis highlighted that after the immersion in follicles medium, the typical peaks ascribable to the gelatin are still visible in the samples after all the timepoints, while after immersion in PBS, these bands could be detected only on the samples immersed for 1 day, as showed in Fig. 9a,b. The explanation on this different behavior in the different media could be related to the different composition of the tested media. In fact PBS is composed by different salts, as NaCl, KCl, Na_2_HPO_4_ and KH_2_PO_4_, while the follicles medium is composed by more reagents (as reported in details in Methods section). Among them, the presence of glucose could be responsible for a mild crosslinking of gelatin, as already reported in literature^45,46^.

### Follicles survival on the scaffolds

**Figure 10:**
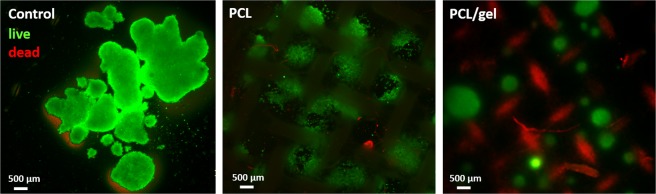
Fluorescence microscope images of LIVE/DEAD assay after 10 days of follicles culture for the control (PET membrane), neat PCL net and PCL/gel net.

Porcine follicles viability was assessed after 10 days of culture on the scaffolds. LIVE/DEAD assay results reported in Fig. 10 showed high number of viable follicles (in green) for PCL, PCL/gel and PET membrane used as control. On PCL net samples, it is possible to notice that the green signal of living follicles is in correspondence of the void of the net structure, demonstrating a preferential location for adhesion and growth. On the other side, on PCL/gel sample it is possible to notice the scaffold structure in red, because the scaffold absorbed the red dye during the staining, giving important information on the position of the follicles on the net structure, in particular, it is interesting to notice that the follicles adhere not only when they are in contact with struts as reported by Laronda *et al*.^28^, but on the entire surface of the fibrous scaffold.

**Figure 11:**
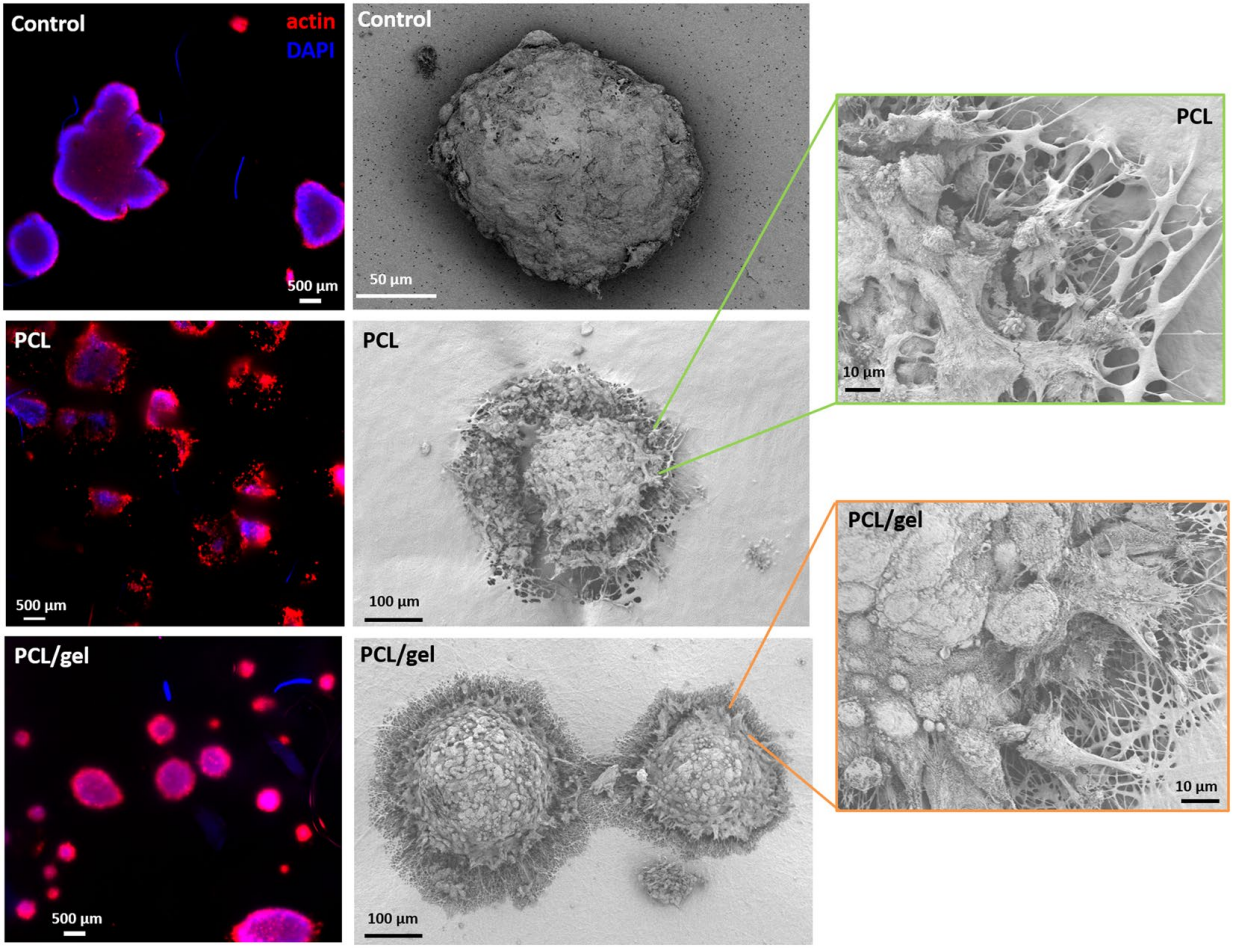
Fluorescence microscope images and SEM micrographs of porcine follicles seeded on electrospun PCL, PCL/gel and control after 10 days from the seeding. Higher magnifications SEM micrographs are reported for PCL and PCL/gel sample to highlight the interaction between follicles and scaffolds. No adhesion points were detected for the control.

The health status of follicles after 10 days of culture was assessed as follows and reported in Table 2: follicles with the oocyte and all the granulosa cells (GCs) viable (dead GCs < 1%), minimally damaged follicles (dead GCs < 5% and vital oocyte), moderately damaged follicles (dead GCs = 5–10% and vital oocyte) and dead follicles (dead GCs > 10% or dead oocyte). This classification was elaborated by the authors considering the novelty of the evaluation of follicles status on a scaffold. Results showed that starting from a comparable number of seeded follicles, after ten days high number of healthy follicles were detected for both scaffolds, respect to the control; oocytes were vital in all analyzed follicles. The low number of follicles detected on the control could be explained by the lack of adhesion between the follicles and the PET substrate, as showed in Fig. 11. Between PCL and PCL/gel scaffolds could be noticed that the PCL/gel shows the highest values of viable follicles, comparable with the number of the seeded, confirming its suitability as substrate to improve follicles adhesion and growth.

**Table 2:** Health status of follicles among PCL, PCL/gel and control (PET membrane) samples after 10 days culturing using the Kruskal-Wallis test followed by Dunn**’**s test.

Scaffold	Dead GCs* ≤ 1%	Dead GCs < 5%	Dead GCs = 5–10%	Dead GCs > 10%	*p*-value
	n (%)	n (%)	n (%)	n (%)	
PCL	187 (76.64%)	32 (13.11%)	11 (4.51%)	14 (5.74%)	<0.001 (PCL vs. PCL/gel)
PCL/gel	295 (90.21%)	22 (6.73%)	6 (1.84%)	4 (1.22%)	0.119 (PCL vs. Control)
Control	39 (88.64%)	3 (6.82%)	1 (2.27%)	1 (2.27%)	0.766 (PCL/gel vs. Control)

*GCs: granulosa cells.

Morphological analysis of the follicles after 10 days of seeding was performed by staining actin filaments (indicated in red in Fig. 11) and cells nuclei (indicated in blue in Fig. 11). Additionally, also SEM analysis was used to evaluate the interaction between the follicles and the scaffolds. The SEM is not a standard technique used for the evaluation of follicles morphology and interactions, for this reason it was needed an optimization of the fixation process in order to investigate the samples. SEM micrographs reported in Fig. 11 show that all the follicles maintain their surface structure and spheroidal shape, characteristic of healthy follicles. For both the electrospun scaffolds, interactions between follicles and the fibers were detected, as reported in Fig. 11. No adhesion points were detected for the control samples, in which the follicles were seeded on PET substrate. Between the two scaffolds, PCL/gel seems to be able to better support the follicles adhesion, to preserve the follicles spheroidal shape and to enhance interactions between follicles.

**Figure 12:**
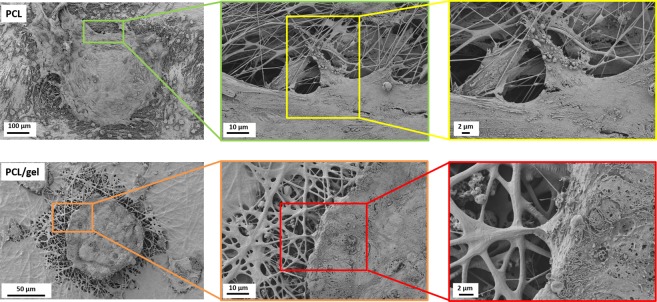
SEM micrographs of porcine follicles seeded on the PCL/gel and PCL electrospun net after 30 days from the seeding. Magnifications and scale bars are indicated inside the micrographs and they were adapted to the optimized view of the follicles, which had different size.

Having in mind the translational purpose, porcine follicles were seeded and cultured on the electrospun PCL and PCL/gel nets for 30 days. SEM analysis was used to investigate the follicles morphology and the interactions between the follicles and the scaffolds. Results reported in Fig. 12 show that also for a longer time point, the follicles survived and maintained their characteristic spheroidal shape for both the scaffolds. The same type of interactions between the follicles and the fibers already noticed in the previous time point (and reported in Fig. 11) are evident in both samples and showed in details in the SEM micrographs at higher magnification in Fig. 12.

**Figure 13:**
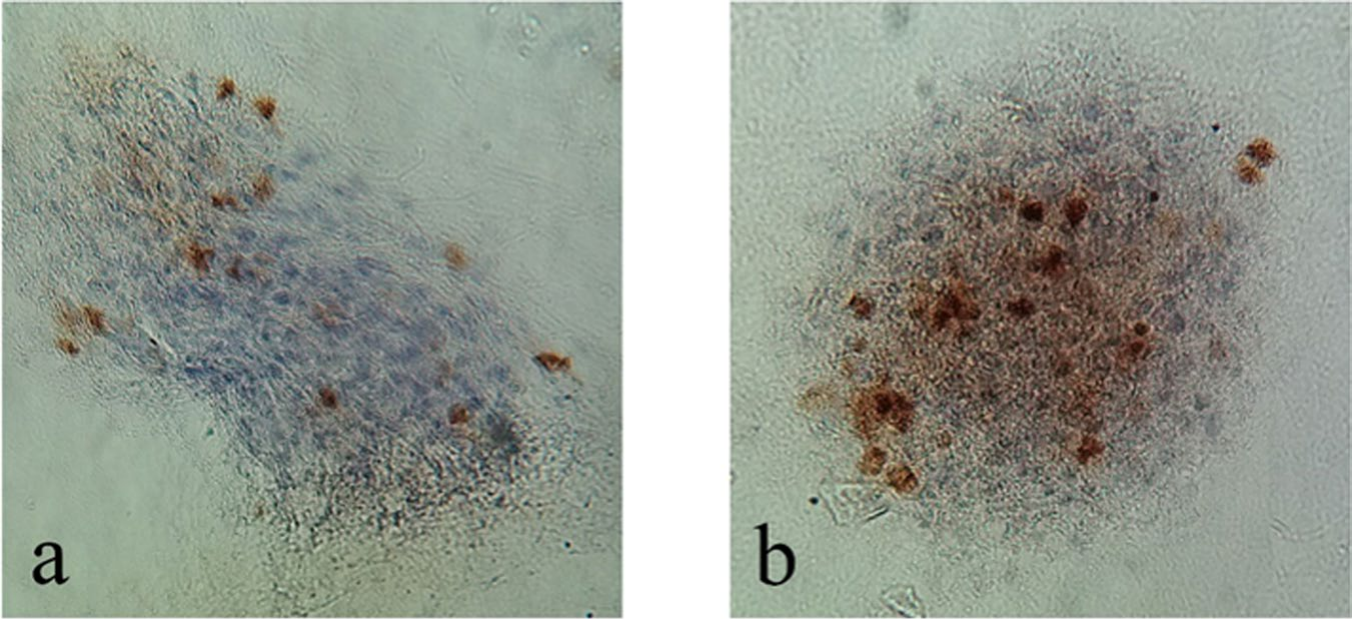
Immunohistochemical (Labelled for Ki67) evaluation of follicles proliferation after 10 days in a) PCL and b) PCL/gel net scaffolds.

Follicles proliferation was evaluated qualitatively by using Ki67 as a proliferation marker and results are reported in Fig. 13. Follicles cultured in both electrospun PCL and PCL/gel scaffolds were positive for Ki67, confirming the suitability of both scaffolds for follicles proliferation, in comparison to neat PCL scaffolds, the signal for Ki67 of follicles in PCL/gel net were more intense.

Estradiol concentrations after 2 days of follicle culture were 362.0 ± 36.8 pmol/L for PCL and 409.0 ± 46.7 pmol/L in case of PCL/gel and increased to 940.3 ± 2.5 pmol/L for PCL and 1024.8 ± 67.5 pmol/L for PCL/gel at day 10. The measured values of progesterone levels after 2 days of culture were 1.9 ± 0.7 and 8.4 ± 3.7 nmol/l in PCL and PCL/gel seeded scaffolds, respectively. After 10 days of culture, the measured values increased and they were 6.5 ± 0.3 and 10.3 ± 6.6 nmol/l, for PCL and PCL/gel scaffolds, respectively. Such increases in estrogen and progesterone productions in spite of follicles and granulosa cells (progesterone producing cells) loss, can clearly confirm the follicles growth. On the other hands positive association between follicle size and estrogen as well as progesterone production has been reported in human and porcine^47,48^.

## Discussion

Natural polymers like alginate, gelatin are mainly used for scaffold fabrication and for follicles culture because of their biocompatibility, biodegradability, processability in hydrogel or beads and presence of biofunctional molecules which usually enhance cells-biomaterial interaction, improving cell adhesion, migration and differentiation. On the other hand, they usually have poor mechanical properties, not comparable to the native tissue, exhibiting difficulties in handling the sample during the implantation. Most of them are enzymatically biodegradable, making the control of degradation rate challenging^15^. Hydrogels of natural polymers used for 3D follicles culture should also have an optimal stiffness value to avoid follicles apoptosis, due to limitation of follicles expansion^43^. Synthetic polymers have not been widely investigated for follicles culture, but they are widely used for scaffold fabrication for tissue engineering and regenerative medicine purpose^16^. They are biocompatible and their mechanical properties and degradation rate could be triggered according to their clinical applications. On the other hand, they lack essential molecules for cell adhesion and proliferation and for some polymers the degradation products in high concentration could cause local inflammation in *in vivo* model^15^. The use of blends constituted by natural and synthetic polymers represents a key solution which could combine the benefits of both polymeric classes, in order to obtain the ideal substrate to support follicles adhesion and growth. In the present paper, for the first time the use of PCL and its blend with gelatin was investigated for the follicles culture.

Besides the biomaterials selection, the scaffold morphology should be similar to the ovarian healthy tissue, in order to be able to host follicles culture and to allow adhesion, growth and maturation. In a recent work Chiti *et al*.^42^ highlighted the relevance of the replication of the dense fibrous ultrastructure, composed by nanometric collagen fibers of the human ovarian cortex. In particular, they reported the importance of using the human ovarian tissue architecture as reference standard for artificial ovary design. For this reasons, in the present work the electrospinning technique was used for the fabrication of scaffolds with a hierarchical structure composed by submicrometric fibers, with nanoporosities arranged in nets with macropores. Another important issue related to the scaffolds design and hierarchical structure is that it can promote the *in vivo* vascularization of the scaffolds^49,50^. Besides the novelty introduced by the biomaterials selection and the scaffold fabrication technique, also the use of porcine follicles represents an innovative approach for the design of artificial ovary.

In fact, in literature, all the *in vivo* studies were performed in mice models with reduced transplantation time. The use of large animal models and future translational aims require slow degradation rate of the scaffolds compatible with the duration of follicle maturation. The use of porcine models presents numerous advantages respect to the mouse model, as length of follicles maturation period more comparable to the human respect to murine model, with less ethical concerns.

Promising results were obtained for both the electrospun PCL and PCL/gel scaffolds in terms of reduced, nearly absent, follicles loss during the seeding and follicles survival after longer time points, respect to the literature. By comparing the performance of both scaffolds, PCL/gel fibers showed better results in terms of lack of follicles loss, preservation of spheroidal shape and higher number of adhesion points with the electrospun mats. The commercial available PET substrate suitable for cell culture, used as control, resulted completely not suitable to allow follicles adhesion showing high values of follicles loss.

In conclusion, scaffold morphology has an important role in the design of artificial ovary. The present study demonstrates that even with the optimized morphology, the appropriate biomaterial selection plays a pivotal role for the follicles adhesion and preservation of their spherical morphology. Further studies are still on-going to investigate in details and improve scaffold-follicles interaction at the interface. As further development, it would be advantageous to have methods to determine and predict oocyte functionality *in vitro* within a given artificial ovary; like linking Anti-Müllerian hormone secretion by follicles with functional oocyte viability in *in vitro* fertilization cycles^51,52^.

## Methods

### Solutions for electrospinning

Poly(epsilon-caprolactone) (PCL) (80 kDa, Sigma Aldrich, Germany) was dissolved in glacial acetic acid (AA) (VWR, Germany), according to a previous protocol developed by the same authors^21^. Briefly, PCL was dissolved in AA (20%w/v), stirred overnight and put in the ultrasonic bath for 1 hour before the electrospinning process. For the blended samples, gelatin (gel) (type A, Sigma Aldrich, Germany) was put with PCL in a mixture of glacial acetic acid and formic acid (ratio 9:1). Briefly, a solution containing 10%w/v of PCL and 5%w/v gelatin was prepared, stirred for 3 hours and directly processed with the electrospinning.

### Scaffolds fabrication

The electrospinning parameters were optimized and set as follows. For neat PCL scaffolds, the applied voltage was 15 kV, the distance needle-target was 11 cm, the polymeric solution flow rate was 0.4 ml/h and the needle diameter was 23 G. For the blend PCL/gel the parameters were kept constant beside the solution flow rate that was increased to 0.6 ml/h. The process was performed in a controlled chamber at 25 °C with 25% relative humidity (EC-CLI, IME Medical Electrospinning, the Netherlands).

### Scaffolds characterization

Scaffolds morphology was investigated by using a scanning electron microscope (Zeiss, Germany), before the analysis the samples were gold sputtered by using Quorum Technologies (UK) Q150T S gold sputter. Light microscopy (Leica M50, Leica, Germany) was used to assess macroscopic changes on the sample surface and pattern. The calculation of the average fiber diameter and the analysis of the porosity were performed by using ImageJ and its plugins (DiameterJ and OrientationJ)^53^. Chemical characterization of the obtained scaffolds was assessed with ATR-FTIR analysis (IRAffinity-1S, Shimadzu, Japan). The measurements were performed with a resolution of 4 cm^−1^, in the range of 4000–600 cm^−1^, with 32 scans. Degradation tests were performed by immersing the samples in Phosphate Buffered Saline (PBS) at 37 °C and in the follicles medium (McCoy’s 5a medium, LifeTechnologies) at 38.5 °C for fixed time points (1, 10 and 30 days of immersion). During the assessment, possible changes in the pH of the solution were monitored by using a bench pHmeter (Mettler Toledo). After the degradation, scaffolds morphology was assessed with SEM, and possible chemical alterations were investigated with ATR-FTIR spectroscopy.

### Follicles extraction and seeding onto the scaffolds

Half of a porcine ovary, collected from the slaughterhouse (registration number DE 09 562 00 38 21; district veterinary office Erlangen-Höchstadt) was washed with cold Dulbecco’s Phosphate Buffered Saline (DPBS, D8661, Sigma-Aldrich) and dissected into 4–5 mm^3^ pieces using scalpers. After two times washing with cold DPBS, the pieces were transferred into a 50 mlcc falcon tube containing 2.5 mlcc lysis buffer. The lysis buffer was prepared by adding 1.5 mg Collagenase (C2674, Sigma-Aldrich) into cold DPBS (final concentration of 0.6 mg/ml). The sample was incubated in a 37 °C water bath for 70 minutes. Every 10 minutes, the tube was gently shaken for about 20 seconds. After the incubation, in order to stop further enzymatic digestion, 10 ml of cold DPBS was added into the tubes and the tube was shaken vigorously. The content of the tube was transferred into a petri dish and the follicles were collected under an inverted microscope with the magnification of 250X. Only non-ruptured follicles with intact morphology and diameter ≤175 µm were selected for cultivation. The collected follicles were washed 3 times in sterile DPBS and transferred into the culture well; different diameters and therefore developmental stages (primordial to preantral stages) were equally distributed to the PCL, PCL/gel and control probes. The follicles were cultured in serum free culture medium (modified version of the medium used by Telfer *et al*.^54^: McCoy’s 5a medium (LifeTechnologies) containing 20 mM HEPES buffer (Gibco), 3 mM L-Glutamine (Gibco), 0.1% BSA (fraction V, CarlRoth), 0.1 mg/ml of each Penicillin and Streptomycin (Sigma-Aldrich), Fungizone (Gibco; final Amphotericin B concentration: 2.5 µg/ml), ITS solution (Sigma-Aldrich; final concentrations: Insulin 8 µg/ml; Transferrin 4.4 µg/ml; Selenium 4 ng/ml), 50 µg/ml Ascorbic acid (Sigma-Aldrich) and 0.272 IE rFSH (Gonal-f, Merck). Before the seeding the electrospun scaffolds were fixed on sample holders (CellCrown^TM^ 24, Sigma Aldrich) and put in 24 well plates. The samples in the multiwells were disinfected by UV light irradiation for 30 minutes before the seeding. CellCrown™24 inserts with 0.4 micron PET membrane (Scaffdex, Finland) were used for follicles seeding as control samples. The follicles were cultured for 10 days at 38.5 °C in humidified air with 5% CO_2_ and half the medium being removed and replaced every second day. Equal numbers of follicles were cultured in the PCL, PCL/gel and control culture conditions and the experiments were conducted three times for each culture condition. Number of follicles per sample was 150 +/−100; each culture time the numbers were equal in all three culture wells.

### Follicles morphology on the scaffolds

The morphology of the follicles seeded on the scaffolds was assessed by SEM analysis after 10 and 30 days of culture. In particular, the samples were fixed by using a solution containing glutaraldehyde, paraformaldehyde, sucrose, and sodium cacodylate trihydrate (Sigma Aldrich, Munich, Germany). Subsequently, the samples were dehydrated after immersion in a series of ethanol solutions. Before SEM analysis, the samples were dried by using a critical point drier (Leica EM CPD 300, Leica, Germany) and gold sputtered.

The LIVE/DEAD™ Viability/Cytotoxicity Kit (ThermoFisher Scientific, Germany) was used to examine the viability of incubated follicles; the kit allows for the simultaneous determination of live and dead cells. Live cells have a ubiquitous intracellular esterase activity, converting the almost non-fluorescent cell-permeating calcein acetoxymethyl (AM) to calcein. Calcein is well retained within live cells, producing an intense uniform green fluorescence in live cells (ex/em ~495 nm/~515 nm). Ethidium homodimer-1 (EthD-1) is only able to enter cells with damaged membranes; it produces a bright red fluorescence upon binding to nucleic acids (ex/em ~495 nm/~635 nm). To perform the viability assay, the LIVE/DEAD working solution (2 µm calcein AM and 4 µM EthD-1) was prepared by adding 10 µl of the 2 mM EthD-1 stock solution and 5 µl of the supplied 4 mM calcein AM stock solution to 10 ml of sterile Dulbecco’s phosphate-buffered saline (D-PBS). The samples were washed in D-PBS three times before adding the working solution directly to the samples. Samples and working solution were left to incubate for 30 minutes in a dark place at room temperature and then evaluated under a Zeiss fluorescence microscope (IM 35, Zeiss, Oberkochen, Germany).

Follicles morphology was evaluated by using Rhodamine Phalloidin (ThermoFisher Scientific, Germany) and DAPI (ThermoFisher Scientific, Germany). The samples fixation was performed by using a solution containing PIPES buffer, ethylene glycol tetra acetic acid (EGTA), polyethylene glycol (PEG), paraformaldehyde, PBS and sodium hydroxide (Sigma Aldrich, Germany), after washing the samples with PBS, the samples were immersed in a permeabilization buffer for intracellular staining and subsequently rhodamine phalloidin was added to each well in a ratio 8 µL/mL and incubated at 37 °C for 1 hour. After the removal of the dye, the samples were intensively washed with PBS and DAPI was added to each well in the ratio of 1 µL/mL. After the removal of the dye the samples were washed in PBS and analyzed with a fluorescent microscope (Axio Scope A1, ZEISS, Germany).

### Follicles proliferation and hormone secretion

The scaffolds were put on glass holders and fixed with an alcoholic fixation solution to prepare them for Ki-67 immunostaining. After rehydration, antigen retrieval was done with heat at pH 6 with target retrieval solution (Dako, Germany, Waldenbronn) according to the manufacturer’s instructions. The immunostaining was conducted on an automated immunohistochemistry staining system (intelli Path FLX, Biocare, USA, Ca, Pacheco) with a mouse anti Ki-67 monoclonal antibody (Zytomed, mouse anti Ki-67 (MSK018) Dako, Germany, Waldenbronn) diluted 1:200 and incubated for 30 minutes at room temperature. For detection the Zytochem-plus HRP kit was used (Zytomed, Germany, Berlin) with incubation times of 20 minutes each from the biotinylated secondary antibody and the Streptavidin conjugate and stained for 12 minutes with a DAB substrate (DAB Substrate Kit, Zytomed, Germany, Berlin) and counterstained with haematoxylin (Dako, Germany, Waldenbronn).

Estradiol and Progesterone concentrations during follicle cultivation with PCL and PCL/gel were evaluated by analyzing removed culture medium during replacement; supernatants from day 2 and day 10 were analyzed in duplicates by using the Access Estradiol and Progesterone Reagent Packs (Beckman Coulter, USA, Ca Brea) with the UniCel DxI 600 Access Immunoassay System (Beckman Coulter, USA, Ca Brea), according to the manufacturer.

### Decellularization porcine ovarian tissue

Decellularized porcine ovarian tissue was obtained by following a modified version of the protocol used by Laronda *et al*.^55^. Briefly, fresh porcine ovaries were cut to 5 × 4 × 0.5 mm^3^ pieces (cortex and medulla). Decellularization was performed via incubation for 38 hours in 2 ml of a 0.1% sodium dodecyl sulfate (SDS; Sigma, USA, MO, St. Louis) solution (SDS in deionized water) and rotation at room temperature (22 ± 1 °C). Then, treated samples were washed three times in deionized water while rotating at room temperature (22 ± 1 °C); duration for each washing step was 2 hours. In order to perform the morphological assessment by using SEM analysis, the samples were processed as previously described. Briefly, the samples were fixed (see above for the composition of fixation solution) and dehydrated with ethanol series. The samples were then freeze dried and gold sputtered before the SEM analysis.

### Statistical analysis

After performing Kolmogorov Smirnov test, the Kruskal-Wallis test followed by Dunn’s test was used for comparison of follicles health status among the groups. For comparison of the quantitative parameters (such as pH) we applied ANOVA test and post-hoc Tukey as the follow-up test. p‐values < 0.05 were considered significant. SPSS V.16 software was used for the statistical analysis.

## Data Availability

The authors declare that all data supporting the findings of this study are available within the paper.
